# Isolation and Chemical Characterization of Chondroitin Sulfate from Cartilage By-Products of Blackmouth Catshark (*Galeus melastomus*)

**DOI:** 10.3390/md16100344

**Published:** 2018-09-20

**Authors:** José Antonio Vázquez, Javier Fraguas, Ramón Novoa-Carvallal, Rui L. Reis, Luis T. Antelo, Ricardo I. Pérez-Martín, Jesus Valcarcel

**Affiliations:** 1 Group of Recycling and Valorisation of Waste Materials (REVAL), Marine Research Institute (IIM-CSIC), Eduardo Cabello, 6. Vigo, 36208 Galicia, Spain; xavi@iim.csic.es; 2 Group of Food Biochemistry, Marine Research Institute (IIM-CSIC), Eduardo Cabello, 6. Vigo, 36208 Galicia, Spain; ricardo@iim.csic.es; 3 3B’s Research Group—Biomaterials, Biodegradables and Biomimetics, University of Minho, Headquarters of the European Institute of Excellence on Tissue Engineering and Regenerative Medicine, AvePark, Barco, 4805-017 Guimarães, Portugal; ramon.novoa@i3bs.uminho.pt (R.N.-C.); rgreis@i3bs.uminho.pt (R.L.R.); 4 ICVS/3B’s—PT Government Associate Laboratory, 4805-017 Braga/Guimarães, Portugal; 5 The Discoveries Centre for Regenerative and Precision Medicine, Headquarters at University of Minho, Avepark, Barco, 4805-017 Guimarães, Portugal; 6 Group of Bioprocess Engineering, Marine Research Institute (IIM-CSIC), Eduardo Cabello, 6. Vigo, 36208 Galicia, Spain; ltaboada@iim.csic.es

**Keywords:** chondroitin sulfate production, cartilage *Galeus melastomus* by-products, sulfation patterns, process optimization, molecular weight glycosaminoglycans determination, bycatch waste management

## Abstract

Chondroitin sulfate (CS) is a glycosaminoglycan actively researched for pharmaceutical, nutraceutical and tissue engineering applications. CS extracted from marine animals displays different features from common terrestrial sources, resulting in distinct properties, such as anti-viral and anti-metastatic. Therefore, exploration of undescribed marine species holds potential to expand the possibilities of currently-known CS. Accordingly, we have studied for the first time the production and characterization of CS from blackmouth catshark (*Galeus melastomus*), a shark species commonly discarded as by-catch. The process of CS purification consists of cartilage hydrolysis with alcalase, followed by two different chemical treatments and ending with membrane purification. All steps were optimized by response surface methodology. According to this, the best conditions for cartilage proteolysis were established at 52.9 °C and *pH* = 7.31. Subsequent purification by either alkaline treatment or hydroalcoholic alkaline precipitation yielded CS with purities of 81.2%, 82.3% and 97.4% respectively, after 30-kDa membrane separation. The molecular weight of CS obtained ranges 53–66 kDa, depending on the conditions. Sulfation profiles were similar for all materials, with dominant CS-C (GlcA-GalNAc6S) units (55%), followed by 23–24% of CS-A (GlcA-GalNAc4S), a substantial amount (15–16%) of CS-D (GlcA2S-GalNAc6S) and less than 7% of other disulfated and unsulfated disaccharides.

## 1. Introduction

Glycosaminoglycans (GAGs) are linear polymers consisting of repeating *O*-linked disaccharide units present in the extracellular matrix or at the cell surface of most animal tissues. GAGs’ ability to interact with proteins is behind their involvement in important cellular events such as cell proliferation, differentiation and migration [1]. As a consequence, GAGs have shown a range of biological activities and are actively explored in the pharmaceutical and tissue engineering fields [2,3,4].

Most GAGs are commercially produced from terrestrial animals, but can also be isolated from marine organisms. Because of the different evolutionary pathways followed by each group of organism, marine and terrestrial GAGs are different, mainly in terms of molecular weight and sulfation [5,6]. Both chemical characteristics are particularly important for the biological functionality of GAGs. In some cases, a specific sequence of saccharides is required for biological activity, for example a pentasaccharide in heparin is responsible for its anticoagulant properties. However, interactions between GAGs and proteins are generally not so specific and seem to be rather influenced by charge density and the presence of particular sulfated units [7]. Thus, sulfated marine GAGs probably represent the most interesting molecules from a therapeutic perspective, chondroitin sulfate (CS) in particular [5].

CS is composed of glucuronic acid (GlcA) and *N*-acetyl galactosamine (GalNAc) linked via alternating β-(1→4) and β-(1→3) glycosidic bonds, and each disaccharide unit can be sulfated at different positions. Marine CS were reported to have different activities such as antiviral, anti-metastatic, anticoagulant and anti-inflammatory activities [1,8,9], to provide signaling properties to cartilage engineering constructs and to improve their mechanical performance [10,11] and to promote neurite outgrowth when hybridized with dermatan sulfate [12]. These biological activities are associated in many cases to the abundance and kind of sulfation pattern, and both are characteristic of each organism [13]. Accordingly, exploration of new sources of CS holds potential to expand the possibilities of different sulfation configurations that may have improved therapeutic properties.

Because of the current overexploitation of marine resources and associated challenges for the fishing industry, new marine sources should be evaluated from the point of view of sustainability. In this regard, valorization of fish by-catch represents an interesting alternative to current discard practices. Within the wide scope of this approach, CS from fish cartilage has been identified as one of the most suitable products for valorization due to its high price and relatively low environmental impact [14]. A number of species of cartilaginous fish have little economic value; however, under current European Union legislation, fishing vessels must keep on board these non-target species if they are subject to quota regulations [15]. This is the case of the blackmouth catshark (*Galeus melastomus*), a shark common in the Northeastern Atlantic Ocean and the Mediterranean Sea. Being abundant, *G. melastomus* is incidentally caught by commercial trawl fisheries [16,17].

Blackmouth catshark appears therefore as a sustainable source of CS, a valorization product that could increase the economic value of this species and serve as an incentive to abandon discard practices. Furthermore, the characteristics of CS extracted from *G. melastomus* are largely unknown, since only one previous report has described some structural features and properties of this material [18]. Important characteristics of CS such as molecular weight and disaccharide composition have not been quantitatively evaluated and, to the best of our knowledge, remain unknown.

In the present work, we aim to fully characterize CS isolated from blackmouth catshark under optimal conditions, defined by response surface methodology. In line with the sustainability principles that guide this study, hydrolysis of cartilage is carried out by enzymatic methods, instead of conventional chemical treatments with toxic guanidine hydrochloride and concentrated alkali [19]. Finally, time-consuming chromatographic separations for CS purification are replaced with more straightforward ultrafiltration-diafiltration techniques.

## 2. Results and Discussion

The average (±confidence interval) proportion of cartilage in the analyzed individuals amounted to 6.80 ± 0.40% (percentage of total weight) with a moisture content of 67.9 ± 3.7%. Chemical composition of cartilage, as % of dry weight, results in 55.0 ± 0.9% protein, 37.0 ± 1.8% ash, 2.0 ± 0.5% fat and 6.0 ± 0.3% carbohydrates. These values are in agreement with the proximal composition found for *Scyliorhinus canicula* cartilage [20].

### 2.1. Hydrolysis of Cartilage by Enzyme Proteolysis

The first step for the isolation of glycosaminoglycans was the enzymatic digestion of cartilage from heads, central skeletons and fins of *G. melastomus* by-products. The enzyme selected was alcalase, a well-known endoprotease with excellent capacity to hydrolyze several marine substrates [21,22,23,24], including cartilage from other fish species [25,26]. The kinetics of enzyme hydrolysis were performed under the experimental conditions defined in Table 1 and the Materials and Methods Section.

**Figure 1:**
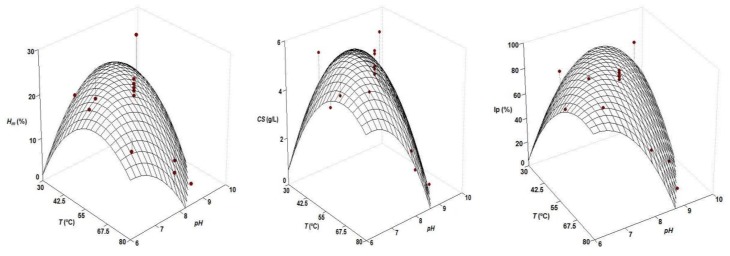
Experimental data and theoretical surfaces obtained from the equations shown in Table 1 describing the joint effect of *pH* and T on the maximum hydrolysis (*H_m_*), chondroitin sulfate (*CS*) concentration and CS purity (*I_p_*) generated by alcalase hydrolysis of cartilage by-products of *G. melastomus*.

The kinetic data of hydrolysis, with hyperbolic trends, were perfectly modelled by the Weibull equation [23], achieving determination coefficients ranging from 0.980–0.998 and complete statistical significance of kinetic parameters (data not shown). One of those parameters, maximum hydrolysis (*H*_m_), was chosen as the response variable to study the joint influence of *pH* and temperature (*T*) on alcalase hydrolysis. The concentration of chondroitin sulfate (*CS*) from samples of the hydrolysates extracted at 0.5 M NaOH/1 *v* EtOH and the index of CS purity (*I_p_*) were also determined. In all cases, the predicted response surfaces were very similar with clear convex shapes (Figure 1). The second order equations that calculated those theoretical surfaces are summarized in Table 2.

Statistically, the consistency of models was always validated after overcoming the *F*1 and *F*2 ratios from *F*-Fisher tests (data not shown). The numerical derivation of equations to obtain the optimal values of both variables, maximizing the response studied, led to the results indicated in Table 3. *pH*_opt_ and *T*_opt_ ranged from 7.06–7.61 and from 47.5–57.8, respectively. In this context, the best conditions to hydrolyze cartilage from *G. melastomus* with alcalase (compromise option as the average of the mentioned intervals) were established at *T* = 52.9 °C and *pH* = 7.31.

### 2.2. Isolation of CS by Chemical Treatments

**Figure 2:**
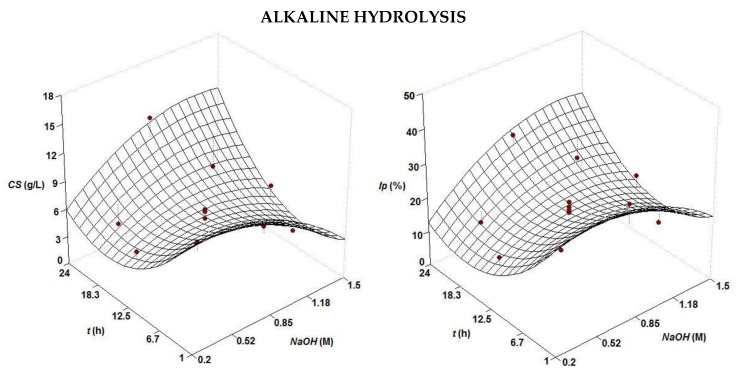

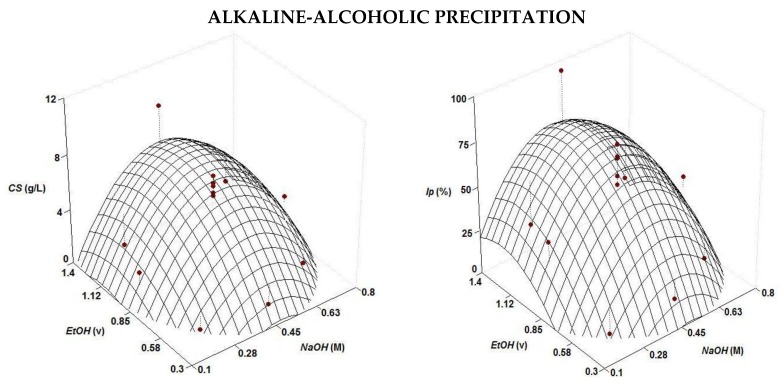
Experimental data and predicted response surfaces by empirical equations summarized in Table 2 corresponding to the combined effect of NaOH and EtOH on the selective treatment of CS from cartilage hydrolysates of *S. canicula.* Responses were *CS* concentration (**left**) and purity index, *I_p_* (**right**).

For the present step, two strategies for improving chondroitin sulfate isolation were evaluated: (1) alkaline hydrolysis to produce CS useful for nutraceutical formulations and (2) selective precipitation of CS in alkaline-alcoholic solutions to yield purer CS useful for medical applications. Initially, hydrolysates of cartilage were produced under the optimal conditions previously defined (*t_h_* = 8 h, *T* = 53 °C, *pH* = 7.3, [alcalase] = 0.5% (*v*/*w*), solid:liquid ratio (1:1), agitation = 200 rpm), in enough amount to perform the two factorial designs of the chemical processing (Table 1). CS concentration and *I_p_* responses (both experimental points and predicted surfaces) from such treatments of the hydrolysates are depicted in Figure 2, and the second order equations are given in Table 2.

The correlation between experimental and predicted was is relatively good with values greater than 0.69, but a lack of fit could be observed in some experimental data (Figure 2). Nevertheless, the consistency of the four cases was confirmed by the values of the F1 and F2 ratios and their comparison to the values from the Fisher F-test (data not shown). In the alkaline hydrolysis, the surfaces showed a heterogeneous concave shape with higher values of CS recovered and purity at short and long times of processing (1 h and 24 h). In both situations, the best concentration of alkalis to maximize the responses was 0.85 M (Table 2). These outcomes were certainly surprising since the expected pattern for the hydrolysis time would be an asymptotic curve (e.g., sigmoid or hyperbolic) rather than the present concave surface observed. No clear assumption could be set to explain this behavior, but a similar parabolic trend for the time of hydrolysis was found in the extraction of antioxidants from surplus tomato crop assisted by microwave [27], the solubilization of collagen from croaker skin by pepsin hydrolysis [28], enzyme hydrolysis of fish processing waste [29] and the production of fish protein hydrolysates [30]. For the NaOH-EtOH treatment, the surfaces were convex domes, with a clear maximum response, in agreement with the results obtained in the precipitation of CS from other cartilaginous fish species [26,31].

Optimal levels of alkalis and ethanol Table 2 were similar, in the case of alcohol, and lower, for NaOH, to those achieved in *Prionace glauca* [26] and *S. canicula* [20]. The purity of CS isolated after enzyme digestion and chemical processing, in the best conditions of operation, were 30% and 75% for alkaline and alkaline-ethanolic treatments, respectively.

### 2.3. Diafiltration for CS Purification

**Figure 3:**
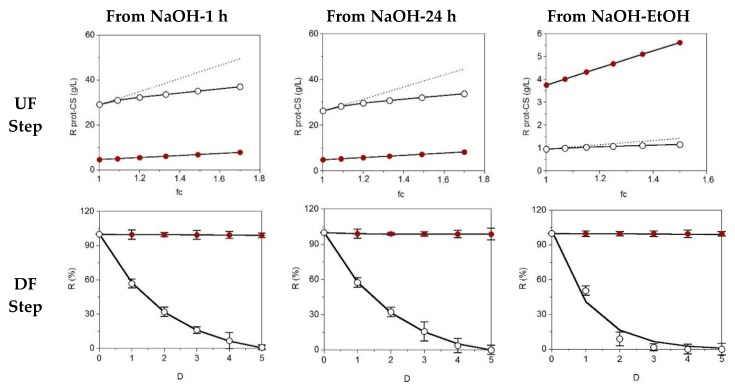
Ultrafiltration (UF) and diafiltration (DF) progress for samples obtained from NaOH (1 h and 24 h of hydrolysis) and NaOH-EtOH treatment. Top: concentration of retained protein (ο) and CS (●) in linear relation with the factor of volumetric concentration (fc) depicting experimental data (points) and theoretical profiles corresponding to a fully-retained solute (discontinuous line). Bottom: progress of protein (ο) and CS (●) retention with the increase of diavolume from DF step (D). Error bars are the confidence intervals (*α* = 0.05; *n* = 2).

The most common protocols for the final purification of glycosaminoglycans are based on chromatography [32,33] or membrane technologies [34,35]. In the present work, we studied the recovery of CS by the ultrafiltration (UF) and diafiltration (DF) steps. Thus, samples obtained by enzyme hydrolysis and subsequent chemical treatments (in all cases, employing optimal conditions) were passed through a membrane of 30 kDa operating in total recirculation. Figure 3 shows the results of the UF-DF stages for the samples generated by selective precipitation (EtOH) and alkaline hydrolysis (NaOH at 1 h and 24 h).

For the case of CS, complete correlation between the experimental and predicted concentration factor was observed, but for the protein fraction, a remarkable amount of this material permeated at the 30-kDa molecular weight cut-off. The DF data were perfectly modelled by the exponential equation [3], obtaining determination coefficients higher than 0.980. The values of the specific retention (*s*), the parameter derived from that equation, indicated the high and low retention of CS and protein, respectively: 0.992 ± 0.017 for CS-NaOH/EtOH, 0.980 ± 0.025 for CS-NaOH-1 h, 0.971 ± 0.023 for CS-NaOH-24 h, 0.090 ± 0.009 for CS-NaOH/EtOH, 0.505 ± 0.021 for CS-NaOH-1 h and 0.523 ± 0.016 for CS-NaOH-24 h. The transmembrane flows during the concentration stage (UF) were maintained, working at 0.8–0.9 bar, at the following levels: 114 ± 21 mL/min, 175 ± 10 mL/min and 182 ± 11 mL/min for the NaOH-1 h, NaOH-24 h and NaOH/EtOH samples, respectively. After drying of retentates, the purities of CS (*I_p_*-values) stood at 81.2%, 82.3% (samples from NaOH treatment) and 97.4% (sample from NaOH/EtOH precipitation). Finally, the yield of CS ranged between 3.5% and 3.7% of wet weight cartilage.

### 2.4. Molecular Weight of CS

**Figure 4:**
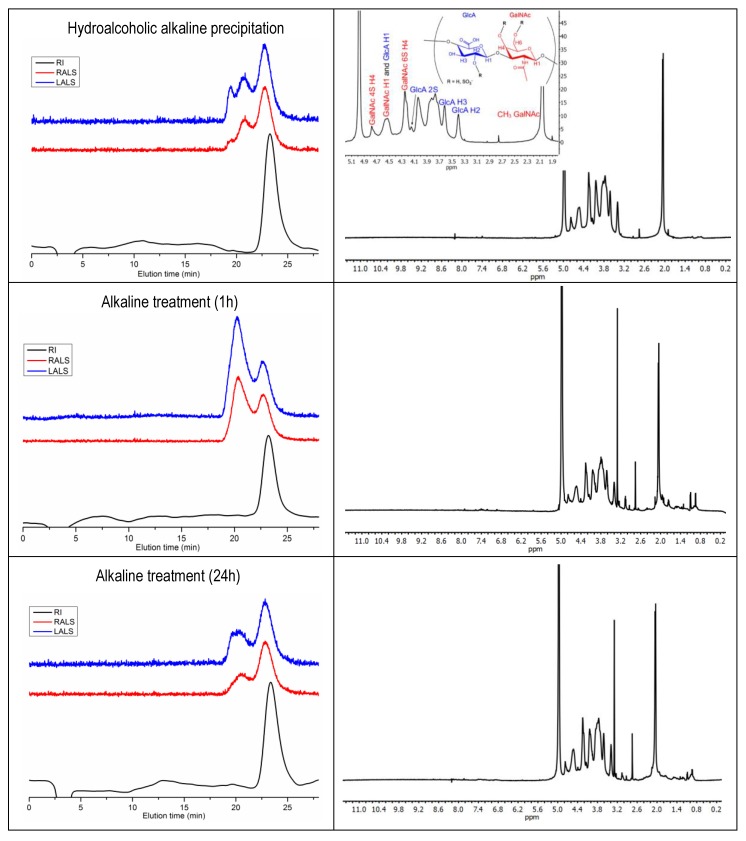
Gel permeation chromatography (GPC) eluograms (**left**) and ^1^H NMR spectra (**right**) of CS extracted from *Galeus melastomus*. Red line: right angle light scattering signal (RALS); blue line: low angle light scattering signal (LALS); black line: refractive index (RI) signal.

The number average molecular weight (Mn) of CS treated with NaOH for 1 h was estimated at 66 kDa; increasing hydrolysis time to 24 h reduced Mn to 53 kDa, comparable to the 55 kDa obtained for hydroalcoholic alkaline precipitation (Table 4). GPC eluograms depicted in Figure 4 show a second peak at low retention times in all samples, which can be observed in the light scattering signals, but is barely visible in the refractive index (RI) trace. This indicates high molecular weight species at a very low concentration. Proteinaceous composition seems unlikely, since additional on-line UV detection from 240–310 nm did not produce any signals. The peak might corresponded to CS aggregates, which have been described in other polyelectrolytes such as chitosan [36,37] or heparin [38], but also other high molecular GAGs occurring in cartilage such as hyaluronan. Unfortunately, hyaluronan presence could not be confirmed by ^1^H NMR because of signal overlap, as discussed in the next section. Regardless of its nature, the low species concentration makes its contribution to CS composition relatively unimportant.

A previous report tentatively estimated the chain length of CS extracted from *G. melastomus* at 27 disaccharide units [18]. This value was calculated from the relative intensities of ^1^H NMR signals of terminal and non-terminal GlcA residues. As the authors recognize, the approximation was only qualitative since other polysaccharide moieties may have contributed to the signal assigned to terminal GlcA, therefore leading to molecular weight underestimation. Indeed, 27 disaccharide units correspond to around 10 kDa (assuming 80% of units mono-sulfated and 15% disulfated), 5–6-times lower than the Mn values reported herein. In other shark species, molecular weight ranges from 31 kDa (unidentified species) [39] to 60 kDa in blue shark (*Prionace glauca*) [40]. In comparison, the molecular weight of CS from *G. melastomus* was relatively high.

### 2.5. Composition of CS

^1^H NMR spectra shown in Figure 4 provide an overview of CS composition. Characteristic CS signals appeared at 2.05 ppm, corresponding to the acetyl group in GalNAc, and in the region from to 3.5–5 ppm. Additional signals outside this range probably correspond to impurities. Amino acids in particular typically appeared between 0.5 and 1.5 ppm (aliphatic) and 7.0–8.5 (aromatic). The number and intensity of these signals were higher for alkaline treatment after 1 h than after 24 h and decreased to its minimum after hydroalcoholic precipitation. This is in line with CS purity index (*I_p_*) values of 81–82% for alkaline treatment and 97.4% for hydroalcoholic alkaline precipitation.

Additional GAGs present in cartilage could also remain as impurities in the final product, specifically hyaluronan, keratan sulfate (KS) and dermatan sulfate (DS). CS and DS both contain GalNAc in their structure, but GlcA in CS is replaced by its epimer iduronic acid (IdoA) in DS. Characteristic signals of DS at 4.87 ppm (H1 of IdoA) and 3.52 ppm (H2 of IdoA) [41] were barely visible in the alkaline-treated samples, implying possible DS presence in minute amounts. Unlike CS and DS, KS and HA share N-acetyl glucosamine (GlcNAc) in their constitutive disaccharides, instead of GalNAc. Anomeric carbons of GlcNAc (H1) present signals at 4.54 ppm in HA and 4.7 ppm in KS [41]. Small amounts of KS can be seen in alkaline-treated samples (Figure 4). The absence of DS and KS signals in the hydroalcoholic precipitated samples indicates that 1.4–1.16 volumes of ethanol used here were capable of separating these GAGs from CS. This agrees with previous reports, which found that DS and KS precipitation occurred below one and above 1.2 volumes of ethanol, respectively, while CS precipitated above one volume of ethanol [42,43]. In the case of HA, it is not possible to assert its presence because the signal at 4.54 ppm overlapped with those of GalNAc and GlcA (H1).

Beyond contaminating compounds, NMR profiles in Figure 4 appear similar for all samples, indicating that differences in treatments did not impact disaccharide composition. Quantification in NMR is difficult because of signal overlap; however, the percentage of units sulfated in position 4 of GalNAc (CS-A) could be estimated by comparing the signal intensities of the acetyl group in GalNAc (2.05–2.07 ppm) with the singlet at 4.78 (H4 of four sulfated GalNAc) [44]. This resulted in 23–24% of CS-A (Table 4), in agreement with the values obtained by strong anion exchange (SAX)-HPLC. Qualitatively, the strong signal at 4.25 denoted a high percentage of CS-C and the singlet at 4.15 the presence of some two sulfated glucuronic acid.

**Figure 5:**
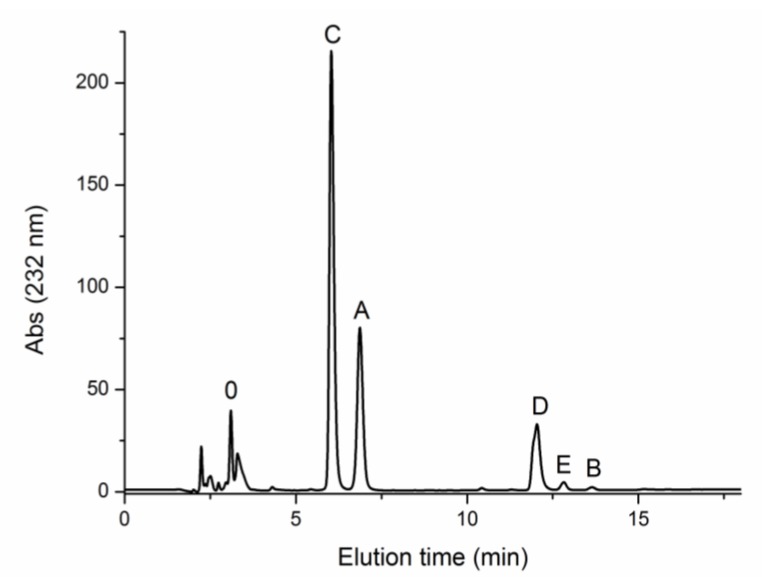
SAX-HPLC chromatogram (UV detection at 232 nm) of CS from *G. melastomus* purified by hydroalcoholic alkaline precipitation after enzymatic digestion with chondroitinase ABC. 0: ΔUA-GalNAc (CS-0); A: ΔUA-GalNAc4S (CS-A); C: ΔUA-GalNAc6S (CS-C); D: ΔUA(2S)-GalNAc6S (CS-D); E: ΔUA-GalNAc4,6S (CS-E); B: ΔUA2S-GalNAc4S (CS-B).

Chromatographic analysis after enzymatic treatment was carried out to complement the information provided by NMR (Figure 5). However, it must be noted that a previous report had shown that treatment with chondroitinase ABC led to 70% hydrolysis after 2.5 h. Even extensive digestion with lyases ABC and C for seven days can only convert 80–85% of the initial polymer to disaccharides [45]. Although this work used an enzyme to substrate ratio 100-times lower than in the present work, it is possible that the hydrolysis performed in the current study was not complete, and disaccharide composition may not fully reflect the proportion in the original polymer. Bearing this in mind, quantitative analysis from chromatography shows that in all cases, the majority of CS disaccharides consisted of CS-C (55%), followed by CS-A (23–24%), with unsulfated CS accounting for only 4% of total CS. Disulfated disaccharides represented 17–18% of total CS, mainly GlcA 2S-GalNAc 6S (CS-D), with only minor quantities of GlcA-GalNAc 4,6S (CS-E) and GlcA 2S-GalNAc 4S (CS-B).

These data showed that CS from *G. melastomus* represents a good source of CS-A and CS-D. Compared to other shark species, CS-C proportion (55%) lied at the high end of the range, typically from 30–60% [40,44,46]. In the case of CS-D, this disaccharide unit is quite uncommon. Cartilaginous fish are its main source, despite the fact that it is not the main disaccharide in fish cartilage. In *G. melastomus*, CS-D accounts for 15–16% of total CS, close to up to 20% reported in *Chimaera phantasma* [46].

While particular applications lie beyond the scope of the current report, CS rich in C units have shown positive results for cartilage regeneration. In vitro, the presence of CS-C appears to enhance chondrocyte proliferation [47,48,49]; favor differentiation of mesenchymal stem cells to chondrocytes and increase extracellular matrix secretion [50,51]. In vivo studies seem to confirm that CS-C improves the ability of hydrogels and scaffolds to repair cartilage lesions [10,52]. Furthermore, CS-C also appears to modulate inflammation to a greater extent than CS-A by reducing NO production and pro-inflammatory cytokines, while increasing the anti-inflammatory cytokine interleukin-10 [53]. These examples serve to illustrate the potential of CS rich in C-units, such as CS from *G. melastomus*.

## 3. Experimental Section

### 3.1. Preparation of Cartilage and Proximal and Analytical Determinations

Cartilage from blackmouth catshark (*Galeus melastomus*) individuals, kindly supplied by Opromar (Marín, Spain), was isolated from the heads, fins and skeletons by treatment with water at 90 °C for 30 min and subsequent manual cleaning. These substrates were crushed and homogenized to ≈1–4 mm and stored at −20 °C until use. The proximal composition of cartilage was determined in triplicate, including moisture, ash, fat, total nitrogen and total protein according to the AOAC protocols [54]. Total carbohydrate content was estimated by subtracting protein, fat, ash and moisture to total sample weight. In CS solutions, total soluble protein (Pr) was determined by the method of Lowry et al. [55]; CS, as glucuronic acid, was quantified by the method of Van den Hoogen et al. [56], according to the modifications of Murado et al. [57]. The CS purity index (*I_p_*), defined as *I_p_* (%) = CS × 100/(CS + Pr), was also calculated in all purification stages.

### 3.2. Factorial Designs and Statistical Analysis

Three experimental designs were performed in the present work to study and optimize: (1) the simultaneous effect of temperature (*T*) and *pH* on the hydrolysis degree of blackmouth catshark cartilage catalyzed by alcalase; (2) the influence of the concentration of NaOH and the time of alkaline hydrolysis on the hydrolysates of cartilage obtained under previous optimal conditions; (3) the effect of NaOH concentration and ethanol volume needed for the selective isolation of CS from cartilage hydrolysates obtained under optimal conditions of hydrolysis. In all cases, the factorial experiments were rotatable second order designs with five replicates in the center of the experimental domains [58]. Codified and natural values for all experimental conditions tested in the factorial designs are summarized in Table 1.

Orthogonal least-squares calculation on factorial design data was used to obtain empirical equations describing the different dependent variables studied (*Y*), each one related to *T* and *pH* for enzymatic hydrolysis and *NaOH* and *EtOH* for CS production. The general form of the polynomial equations is:

(1) $$Y=b_{0}+\sum_{i=1}^{n}b_{i}X_{i}+\sum_{\underset{j>i}{i=1}}^{n-1}\sum_{j=2}^{n}b_{ij}X_{i}X_{j}+\sum_{i=1}^{n}b_{ii}X_{i}^{2}$$

where *Y* is the dependent variable evaluated, b_0_ the constant coefficient, b*_i_* the coefficient of the linear effect, b*_ij_* the coefficient of the combined effect, b*_ii_* the coefficient of the quadratic effect, *n* the number of variables and *X_i_* and *X_j_* the independent variables studied in each case. Student’s *t*-test (*α* = 0.05) was employed to determine the statistical significance of coefficients. The coefficient of adjusted coefficients of determination ($R_{adj}^{2}$) was used to establish goodness-of-fit, and the following mean squares ratios from the Fisher *F*-test (*α* = 0.05) were calculated to define model consistency: *F*1 = model/total error, the model being acceptable when *F*1 ≥ $F_{\mathrm{den}}^{\mathrm{num}}$; and *F*2 = (Model + lack of fitting)/model, the model being acceptable when *F*2 ≤ $F_{\mathrm{den}}^{\mathrm{num}}$. $F_{\mathrm{den}}^{\mathrm{num}}$ are the theoretical values for *α* = 0.05 with corresponding degrees of freedom for the numerator (num) and denominator (den).

### 3.3. Cartilage Enzymatic Digestion

Cartilage was digested with 2.4 L of alcalase from *Bacillus licheniformis* (Novozyme Nordisk, Bagsvaerd, Denmark). The enzyme/substrate ratio was 24 U/kg (1% *v*/*w* of fresh cartilage); the solid:liquid ratio was (1:1); and T and *pH* conditions are defined in Table 1. Hydrolysis was performed in a thermostated reactor as indicated in previous work [20,26]. The progress of enzymatic hydrolysis was determined by the *pH*-Stat method [59], and the non-linear kinetics of hydrolysis degree (*H*, in %) were modelled by the Weibull equation [23]. The maximum degree of hydrolysis (*H_m_*) was the parameter selected from such an equation as the dependent variable for the optimization study.

### 3.4. Chemical Processing of the Hydrolysates

Two kinds of chemical treatments were applied in parallel to the hydrolysates of cartilage obtained by alcalase digestion: (a) alkaline hydrolysis and (b) selective precipitation using hydroalcoholic solutions of NaOH. In the former, NaOH was added to the enzymatic hydrolysates until the concentrations defined in Table 1. The corresponding mixtures were maintained in continuous agitation at 200 rpm and room temperature for the different times studied. At the end of hydrolysis, mixtures were centrifuged at 6000× *g* for 20 min and supernatants neutralized with 6 M HCl. In the second treatment, CS present in the hydrolysates was precipitated by slowly adding NaOH solutions in hydroalcoholic media with different ethanol volumes (Table 1) under medium agitation at room temperature. A concentration of 2.5 g/L NaCl was also present in the mixtures. Suspensions formed were centrifuged (6000× *g*/20 min) after 2 h in agitation and the sediments resuspended in water and neutralized with 6 M HCl.

### 3.5. Purification of CS by UF-DF

A UF membrane of 30 kDa (spiral polyethersulfone, 0.56 m^2^, Prep/Scale-TFF, Millipore Corporation, Burlington, MA, USA) was used to concentrate, desalinate and purify CS solutions obtained in chemical processing. The configuration and operation mode of the membrane system, initial concentration by the UF and then the DF step, were performed according to the description reported by [20]. DF data were modeled by a first-order equation [60], and the specific retention (*s*) parameter from that was calculated for comparative reasons.

### 3.6. Molecular Weight of CS

Absolute molecular weight of CS was determined on a GPC/SEC system (Agilent 1260, Agilent, Waldbronn, Germany) equipped with quaternary pump (G1311B), injector (G1329B), column oven (G1316A), refractive index (G1362A) and dual angle static light scattering (G7800A) detectors. Sample separation was achieved with a set of four columns (PSS, Mainz, Germany): Suprema precolumn (5 µm, 8 × 50 mm), Suprema 30 Å (5 µm, 8 × 300 mm), Suprema 100 Å (5 µm, 8 × 300 mm) and Suprema ultrahigh (10 µm, 8 × 300 mm). A sample volume of 100 µL was injected onto the above system and eluted at 1 mL/min with a solution composed of 0.1 M NaN_3_ and 0.01 M NaH_2_PO_4_ at *pH* 6.6. The column oven and light scattering detector were kept at 30 °C, while the refractive index detector was kept at 40 °C. Both detectors were calibrated with a polyethylene oxide standard (PSS, Mainz, Germany) of 106 kDa (Mp) and a polydispersity index (PDI) of 1.05. Samples and standards were dissolved in the mobile phase solution. Refractive index increments (dn/dc) of 0.110 were calculated from the RI detector response.

### 3.7. CS Composition by ^1^H NMR and SAX-HPLC

Chemical composition of CS was assessed by the combination of NMR and chromatographic techniques.

NMR spectra were recorded on a Bruker DPX 600 (Bruker, Rheinstetten, Germany) operating at 600 Mhz. The temperature was set to 10 °C to avoid overlapping with residual HOD. Samples were dissolved in D_2_O at 1 g/L for ^1^H experiments. Spectral processing was carried out with MestReNova 10.0.2 software (Mestrelab Research, Santiago de Compostela, Spain). Spectra were referenced from the solvent signal.

Disaccharide composition of CS was determined by strong anion exchange (SAX) chromatography after enzymatic digestion with chondroitinase ABC from *Proteus vulgaris* (EC 4.2.2.4., 1.66 U mg^−1^, Product number C2905, Sigma-Aldrich, Saint Louis, MO, USA) at 0.2 U mg^−1^ of CS. The reaction was carried out in 0.05 M Tris-HCl/0.15 M sodium acetate buffer at *pH* 8 and 37 °C. After 24 h, the enzyme was inactivated by heating at 70 °C for 25 min, followed by centrifugation at 12,857× *g*. Supernatants were collected and filtered through 0.2-µm polyethersulfone (PES) syringe filters. Unsaturated disaccharide standards were purchased from Grampenz (Aberdeen, UK) and dissolved in water. Samples and standards were manually injected onto an HPLC system (Agilent 1200) consisting of a binary pump (G1312A), column oven (G1316A) and UV-visible detector (G1314B). Separation was carried out with a Waters Spherisorb SAX column (5 µm, 4.6 × 250 mm, Prod. No. PSS832715, Waters Corp, Milford, MA, USA) fitted with a guard cartridge (Waters Spherisorb, 5 µm, 4.6 × 10 mm) based on a previously reported method [61]. Elution was performed in isocratic mode from 0–5 min with 50 mM NaCl at *pH* 4. The linear gradient was applied from 5–20 min starting with 50 mM NaCl at *pH* 4 and ending with 76% 50 mM NaCl at *pH* 4 and 24% 1.2 M NaCl at *pH* 4. A sample volume of 20 µL was injected onto the system with a flow rate of 1.5 mL min^−1^. Detection was made at 232 nm. An external calibration curve was built with each standard to calculate the amount of disaccharide units in the sample and reported as percentage of weight.

## 4. Conclusions

In the present work, we study CS isolation from *G. melastomus* by initial enzymatic cartilage proteolysis, followed by two different chemical treatments and ending in membrane purification. All steps are mathematically optimized by response surface methodologies. The conditions to maximize CS recovery are established as: 52.9 °C and *pH* 7.31 for enzyme digestion of cartilaginous material; 0.85 M NaOH for alkaline treatment of the EH and 0.45 M NaOH, 1.14–1.16 *v* EtOH for alkaline hydroalcoholic precipitation of the EH; and UF at 30 kDa using at least five diavolumes of water to obtain CS with more than 81–82% of purity (97.4% with NaOH-EtOH solutions). Molecular weights were estimated at 53–66 kDa, relatively high compared to other cartilaginous fish. Sulfation profiles were similar for both chemical treatments, revealing dominant CS-C units (55%), followed by 23–24% CS-A, a substantial amount of CS-D (15–16%) and less than 7% of other disulfated and unsulfated disaccharides.

## Figures and Tables

**Table 1:** Experimental domains and codification of the independent variables in the factorial rotatable designs performed to optimize the enzyme hydrolysis of cartilage and the chemical treatments of the hydrolysates using alkaline or alkaline-hydroalcoholic solutions.

Coded Values	Natural Values					
	Enzyme Hydrolysis		NaOH Treatment		NaOH-EtOH Treatment	
	*pH*	*T* (°C)	*NaOH* (M)	Time (h): *t*	*NaOH* (M)	Ethanol (*v*)
−1.41	6.0	30.0	0.20	1.0	0.10	0.30
−1	6.6	37.3	0.39	4.4	0.20	0.46
0	8.0	55.0	0.85	12.5	0.45	0.85
+1	9.4	72.7	1.31	20.6	0.70	1.24
+1.41	10.0	80.0	1.50	24.0	0.80	1.40

Codification: *V*_c_ = (*V*_n_ − *V*_0_)/∆*V*_n_; decodification: *V*_n_ = *V*_0_ + (∆*V*_n_ × *V*_c_); *V*_c_ = codified value of the variable; ∆*V*_n_ = increment of *V*_n_ per unit of *V*_c_; *V*_n_ = natural value of the variable to codify; *V*_0_ = natural value in the center of the domain.

**Table 2:** Polynomial equations modelling NaOH and time influence in alkaline treatment and NaOH and EtOH in an alkaline-alcoholic precipitation applied to cartilage hydrolysates. Optima values of the independent variables (*NaOH*_opt_, *t*_opt_ and *EtOH*_opt_) are also calculated.

**Treatment**	**Second Order Equations**	$R_{\mathrm{adj}}^{2}$	***NaOH*_opt_ (M)**	***t*_opt_ (h)**
**Alkaline**	*CS* (g/L) = 6.42 + 1.34 *t NaOH* − 0.88 *NaOH*^2^ + 1.68 *t*^2^	0.687	0.85	1 or 24
	*I_p_* (%) = 19.05 + 3.03 *t NaOH* − 2.61 *NaOH*^2^ + 4.37 *t*^2^	0.709	0.85	1 or 24
		$R_{\mathrm{adj}}^{2}$	***NaOH*_opt_ (M)**	***EtOH*_opt_ (*v*)**
**Alkaline-alcoholic**	*CS* (g/L) = 6.56 + 1.91 *EtOH* − 2.39 *NaOH*^2^ − 1.28 *EtOH*^2^	0.742	0.45	1.14
	*I_p_* (%) = 67.0 + 20.90 *EtOH* − 20.06 *NaOH*^2^ − 13.03 *EtOH*^2^	0.710	0.45	1.16

**Table 3:** Polynomial equations modelling *pH* and *T* effects on alcalase hydrolysis of *G. melastomus* cartilage. Adjusted determination coefficients ($R_{\mathrm{adj}}^{2}$) and optimal values of *T* and *pH* (*T*_opt_ and *pH*_opt_) that maximized the dependent variables are also shown.

Second Order Equations	$R_{\mathrm{adj}}^{2}$	*T*_opt_ (°C)	*pH*_opt_
*H_m_* (%) = 22.02 − 5.18 *T* − 4.82 *pH* − 5.56 *T pH* − 4.26 *T*^2^ − 4.44 *pH*^2^	0.801	47.5	7.61
*CS* (g/L) = 5.25 − 0.80 *T* − 1.36 *pH* − 1.20 *T pH* − 0.80 *T*^2^ − 1.16 *pH*^2^	0.796	53.3	7.25
*I_p_* (%) = 85.06 − 11.81 *T* − 23.06 *pH* − 22.76 *T pH* − 10.59 *T*^2^ − 20.02 *pH*^2^	0.890	57.8	7.06

**Table 4:** Molecular weight and disaccharide composition of CS isolated from *G. melastomus* following alkaline hydrolysis (1 h and 24 h) and hydroalcoholic-alkaline precipitation. Mn: number average molecular weight, PDI: polydispersity index; disaccharide composition expressed as the mean% ± the standard deviation; ^1^H NMR, strong anion exchange (^2^SAX)-HPLC.

	Alkaline Hydrolysis 1 h	Alkaline Hydrolysis 24 h	Hydroalcoholic Alkaline Precipitation
Mn	66 kDa	53 kDa	55 kDa
PDI	1.14	1.25	1.26
CS-A (GlcA-GalNAc 4S)^1^	23.9	22.78	23.01
CS-A (GlcA-GalNAc 4S)^2^	23.43 ± 0.23	23.52 ± 0.11	23.77 ± 0.13
CS-C (GlcA-GalNAc 6S)^2^	54.78 ± 0.02	55.11 ± 0.16	54.93 ± 0.36
CS-0 (GlcA-GalNAc 0S)^2^	3.96 ± 0.03	3.92 ± 0.27	4.23 ± 0.55
CS-D (GlcA 2S-GalNAc 6S)^2^	15.75 ± 0.19	15.37 ± 0.00	15.00 ± 0.05
CS-E (GlcA-GalNAc 4,6S)^2^	1.46 ± 0.05	1.46 ± 0.00	1.48 ± 0.01
CS-B (GlcA 2S-GalNAc 4S)^2^	0.61 ± 0.00	0.62 ± 0.01	0.59 ± 0.01
